# High Flow-Rate Sample Loading in Large Volume Whole Water Organic Trace Analysis Using Positive Pressure and Finely Ground Sand as a SPE-Column In-Line Filter

**DOI:** 10.3390/molecules24071426

**Published:** 2019-04-11

**Authors:** Ola Svahn, Erland Björklund

**Affiliations:** Department of Environmental Science and Bioscience, Faculty of Natural Science, Kristianstad University, SE-291 39 Kristianstad, Sweden; erland.bjorklund@hkr.se

**Keywords:** environmental analysis, whole water, trace analysis, SPE, large volume, in-line filter, sand, flow rate, pharmaceuticals, hormones, pesticides

## Abstract

By using an innovative, positive pressure sample loading technique in combination with an in-line filter of finely ground sand the bottleneck of solid phase extraction (SPE) can be reduced. Recently published work by us has shown the proof of concept of the technique. In this work, emphasis is put on the SPE flow rate and method validation for 26 compounds of emerging environmental concern, mainly from the 1st and 2nd EU Watch List, with various physicochemical properties. The mean absolute recoveries in % and relative standard deviations (RSD) in % for the investigated compounds from spiked pure water samples at the three investigated flow rates of 10, 20, and 40 mL/min were 63.2% (3.2%), 66.9% (3.3%), and 69.0% (4.0%), respectively. All three flow rates produced highly repeatable results, and this allowed a flow rate increase of up to 40 mL/min for a 200 mg, 6 mL, reversed phase SPE cartridge without compromising the recoveries. This figure is more than four times the maximum flow rate recommended by manufacturers. It was indicated that some compounds, especially pronounced for the investigated macrolide molecules, might suffer when long contact times with the sample glass bottle occurs. A reduced contact time somewhat decreases this complication. A very good repeatability also held true for experiments on both spiked matrix-rich pond water (high and low concentrations) and recipient waters (river and wastewater) applying 40 mL/min. This work has shown that, for a large number of compounds of widely differing physicochemical properties, there is a generous flow rate window from 10 to 40 mL/min where sample loading can be conducted. A sample volume of 0.5 L, which at the recommended maximum flow rate speed of 10 mL/min, would previously take 50 min, can now be processed in 12 min using a flow rate of 40 mL/min. This saves 38 min per processed sample. This low-cost technology allows the sample to be transferred to the SPE-column, closer to the sample location and by the person taking the sample. This further means that only the sample cartridge would need to be sent to the laboratory, instead of the whole water sample, like today’s procedure.

## 1. Introduction

In environmental organic trace analysis, there is most often a need to enrich the compounds of interest from a large sample volume, where the use of solid phase extraction (SPE) is more or less the standard technique. Extraction and clean-up protocols related to the SPE methodology commonly constitute the bottleneck of the analytical procedure and are estimated to account for 75% of the analysis time [1]. SPE-column manufacturers recommend maximum flow rates in reverse phase SPE (200 mg HLB, Hydrophilic Lipophilic Balanced) and 6 mL from 1 to 10 mL/min, to avoid component breakthrough [2,3,4]. A sample volume of 0.5 L will therefore take between 50 to 500 min to process if such flow rates are applied. These figures are under ideal conditions, without the presence of a possible flow-interfering matrix. The most common commercial technique for loading samples onto SPE columns utilizes a vacuum manifold. However, as the sample volume increases, the matrix content transferred to the SPE column also increases. To prevent clogging, and/if focusing on soluble compounds, samples are often pre-filtered, and in some methods even filtered twice [5]. Even when filtering the samples, the vacuum manifold technique might become tediously slow, especially at the end of a sample load. This problem often remains in spite of reducing the pressure near to the minimum allowed, as recommended by the manufacturer. Often, as is specifically expressed on the 1st and 2nd EU Watch List, substances shall be monitored in whole water samples [6,7]. The reason behind this is that some pharmaceuticals are hydrophobic and lipophilic, and therefore might be associated with particulate matter. As a consequence, for a meaningful monitoring of pharmaceuticals in the aquatic environment, the particulate phase should also be considered. Even so, the majority of all published monitoring studies mainly focus on the water phase [8].

We recently published a short communication presenting a technique, which addresses this obstacle, using positive pressure by compressed air and finely ground sand as an SPE in-line filter [9]. This design facilitates loading larger sample volumes onto SPE columns, as needed in organic trace analysis of environmental surface waters. 

**Figure 1:**
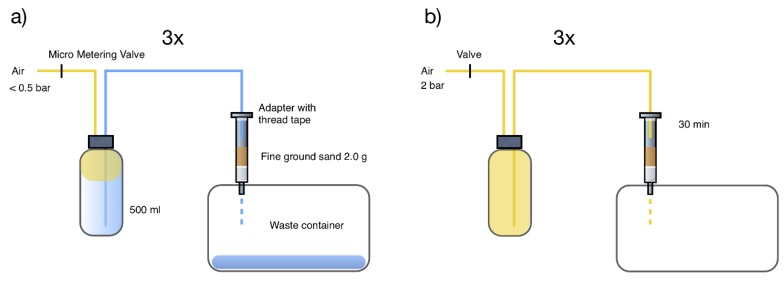
Schematic arrangement of (**a**) the sample loader using compressed air and sand as an in-line filter, and (**b**) the drying procedure of the solid phase extraction (SPE) columns containing analytes, matrix, SPE-polymer, and sand. Adapted from reference [9].

The schematic arrangement of both the sample loader using compressed air, combined with sand as an in-line filter, and the drying procedure of the SPE cartridge containing analytes, matrix, SPE-polymer, and sand is outlined in Figure 1.

In this work, we investigate the possibility of increasing the sample loading flow rate with the purpose of increasing laboratory sample throughput without compromising repeatability and robustness. Flow rate investigations are crucial in determining if this low-cost technique could be applied outside the fully equipped laboratory. We have limited this study to 26 compounds of emerging concern, including all the compounds on the 1st and 2nd EU Watch List with an indicative analytical method of SPE LC-MS-MS (Liquid Chromatography - Tandem Mass Spectrometry) [6,7]. This plethora of compounds possess widely differing physicochemical properties, which will reveal if different properties affect break-through on the hydrophilic lipophilic balanced SPE cartridge at high flow rates. 

**Figure 2:**
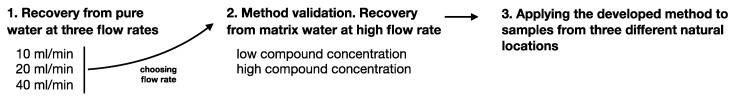
Flow chart illustrating the three main experiments.

Three separate experiments were carried out, and the general construction is outlined in Figure 2. The first experiment was performed in order to determine the maximum flow rate in terms of recovery and repeatability. An increased flow rate raises the need to compare if the produced means are significantly different, or if they are indistinguishable, within the limits of the experimental error. Based on manufacturers recommendations, it was expected that a low flow rate should be less error prone compared to a higher flow rate, and that recoveries would be higher at lower flow rates. Following the manufacturers recommendation, the lowest experimental flow rate value was set to their maximum recommendation at 10 mL/min [4]. 

Next, after determining the flow rate and letting this be the methods foundation, the technique was applied to an artificial matrix water spiked at two concentration levels in order to validate the method for the investigated compounds. Finally, in experiment number three, we investigated the robustness and repeatability of the method by applying it to determine the occurrence and concentrations of the 26 selected compounds in natural river samples and wastewater from three different recipient locations in the county of Scania, Sweden.

## 2. Results

### 2.1. Absolute and Relative Recoveries for the Three Experiments

#### 2.1.1. Recovery from Pure Water at Three Flow Rates

The results of the absolute recoveries of the three investigated flow rates, 10, 20 and 40 mL/min, are presented in Table 1. Mean absolute recovery was found to be 64.2%, 68.2%, and 70.7%, for the three flow rates, respectively, with relative standard deviations (RSDs) of 3.3%, 3.5%, and 4.3%. A total of 17 of the 35 investigated compounds (26 + 9 isotope labeled standards) showed an absolute recovery above 75%, while 8 fell below 50% at 40 mL/min. The corresponding figures for the two other investigated flow rates were 16 above 75% and 8 below 50% at 10 mL/min, and 16 above 75% and 7 below 50% at 20 mL/min. It is also worth pointing out that recoveries are particularly low for amoxicillin, azithromycin, and triallate, with never more than 10% at any condition.

One-way ANOVA was applied to test whether the difference between the flow rate mean results were too great to be explained by a random error. The one-way ANOVA analysis clearly showed that the absolute recovery for 6 (grey marked) of the 35 compounds are significantly different (F-crit. value at 5.14, *p* = 0.05), Table 1. In these cases, there was always a higher mean recovery at the highest flow rate of 40 mL/min. The two antibiotic macrolides azithromycin and erythromycin, represent good examples of this behavior. Three of the significantly different mean values are identified among the group of isotope labeled standards (IS); ciprofloxacin_D8, clarithromycin_D3, and sulfamethoxazole_C6. Finally, the sunscreen ingredient 2-ethylhexyl 4-methoxycinnamate also showed a higher recovery at the highest flow rate. In this respect, it could be noted that adsorption of analytes to sample containers of different types has been investigated in trace analysis and found to be significant for some compounds [10]. 

Each pair of compound-IS combination used to calculate the relative recovery for the three experiments are shown in the second to last column of Table 1**.** Using the IS method, 4, 5, and 4 compounds out of 26 showed a recovery lower than 50% (grey marked) at 10, 20, and 40 mL/min. The mean relative recovery was found to be 83.1%, 76.7%, and 74.1% for the three flow rates, respectively, with an RSD of 6.6%, 3.3%, and 4.5%. There was a trend of generally higher relative calculated concentrations for the two lower flow rates compared to 40 mL/min. The IS method does not compensate to any greater extent for the low absolute recovery previously noted for amoxicillin and azithromycin. 

#### 2.1.2. Method Validation. Recovery Evaluation at High and Low Concentrations in Matrix-Rich Pond Water at the Highest Flow Rate of 40 mL/min

Electrospray ionization processes are known for both ion suppression and ion enhancement, with the former being more common [11]. A diluted humic-rich pond water provided the necessary matrix effect for this to be expressed. The carbon content was found to be 2.0 mg/L in the 1:5 diluted pond water. The absolute recovery results from the former experiment made us confident in picking and proceeding with the highest flow rate of 40 mL/min as the foundation for further method validation. It is noteworthy that not a single value, no matter what chemical functional groups, pointed in the direction of a higher recovery at a lower flow. 

The results for this second set of experiments are presented in Table 2, ranked from the compound with the highest absolute recovery in the pond water to the compound with the lowest absolute recovery. IS compound recoveries are ranked as a separate group at the bottom of Table 2. The absolute amounts of compounds spiked to the 0.5 L sample volume can be found in the last two columns of Table 2. The high value is the same as that applied in the previous set of experiments (Table 1), and the low value represents a spiking level 10 times below that amount. As an example, carbamazepine has a high concentration of 20 ng/L (10 ng/0.5 L) and a low concentration of 2 ng/L (1 ng/0.5 L). 

In the matrix-rich pond water, the mean absolute recovery at the high concentration dropped to 54.7% as compared to 70.7% for pure water (Table 1, absolute recovery C, column 6), but still showed a low mean RSD at 3.6%. In column 4 (Aq-matrix) in Table 2 the difference between absolute recovery in pure water (Table 1, absolute recovery C, column 6) and the pond water (Table 2, high, column 2) are presented for each individual compound. The majority of the compounds expressed a decrease in recovery (indicated as positive values). The decrease ranged from 2–63%, with fluconazole showing the largest difference between pure spiked water and matrix-rich pond water. However, six compounds showed higher absolute recovery in the presence of matrix (grey marked). The increased recovery results in the presence of matrix are particularly pronounced in three cases, namely triallate and the two antibiotics, azithromycin and doxycycline. A moderate enhancement could be noted for erythromycin, while clarithromycin and 2-ethylhexyl 4-methoxycinnamate had very minor increases. One extra experiment; processing un-spiked diluted pond water through the entire sample preparation chain, followed by post-spiking of the compounds to the obtained extract, showed that ion enhancement was indeed the case for these compounds, as blank pond water, processed and analyzed, revealed no traces of any of the investigated compounds.

It was also verified by this experiment that fluconazole suffers from true ion suppression, as post-spiking of the pond water showed a 59% difference in absolute recovery (data not shown) as compared to the pre-spiked pond water with a very similar decrease of 63% (Table 2, Aq-matrix, column 4). The average difference in absolute recovery for the 20 compounds showing ion suppression was 26.4%. Additionally, all 9 IS also suffered from ion suppression with an average difference of 32.9%.

Relative recoveries at low and high concentrations, using the IS calibration method that compensates for matrix interferences, are presented in column 5–8 in Table 2. Sometimes the compensation becomes too high as is the case for azithromycin, doxycycline, triallate and 2-ethylhexyl 4-methoxycinnamate, marked in light grey in Table 2 (high, column 7). A re-calculation using sulfamethoxazole_C6 as an IS (it was the IS with highest absolute recovery), gave a lower and more correct recovery situation for triallate and 2-ethylhexyl 4-methoxycinnamate, but this could not fully adjust for the matrix effect attributed to the two antibiotics, doxycycline and azithromycin. However, both antibiotics were paired with sulfamethoxazole_C6 (final method) before moving into the third experiment below. Native IS would be appropriate to consider regarding these two compounds to achieve better relative recoveries.

#### 2.1.3. Applying the Developed Method to Recipient Samples from Three Different Natural Locations

Results from recipient samples taken from three natural locations in the southern parts of Sweden are found in Table 3. In short, Degeberga represents a sample taken from the Segesholmsån River (average flow 0.6 m^3^/s) downstream of Degeberga village. Degeberga wastewater treatment plant (WWTP) treats wastewater from a population of ca. 1000 people. St Olof represents a sample taken in the Rörums Södra Å River (average flow roughly 0.4 m^3^/s) downstream of St Olof village that has a WWTP treating wastewater from a population of ca. 600 people. Both samples were expected to contain measurable concentrations of a number of compounds occurring in the developed method, though at a low concentration level due to small WWTPs with a great extent of dilution of the wastewater in the river. Pynten is the outlet point of basically undiluted wastewater from the city of Kristianstad’s WWTP into the Hammarsjön Lake. The water has only been transported in a 1500 m long artificial channel from the WWTP to the lake, with only minor inflow of additional surface water. Kristianstad WWTP treats wastewater from ca. 52,000 people. Therefore, at the sampling point Pynten, relatively high concentrations of many of the compounds were expected. A map showing the sampling locations within the county of Scania (Sweden) can be found in Figure S1.

Given the fact that the location near the major city of Kristianstad showed the highest concentrations, the results were ranked based on these concentrations with the compound identified and quantified at the lowest concentration first, as shown in Table 3 (Pynten, column 12).

The IS standards are put alphabetically at the lower part of Table 3 (Degeberga, column 2; St Olof, column 4; Pynten, column 6). The location at Degeberga had a carbon content of 2.6 mg/L in the water and showed the highest absolute recovery values for all IS, except for clarithromycin_D3. Sulphamethoxazole_C6 was almost recovered to 100% in Degeberga. The St Olof water, with a carbon content of 6.2 mg/L, had the second highest absolute recovery values, and last came the water sample from Pynten (Kristianstad), which had a carbon content of 2.4 mg/L. All three sample locations showed low RSDs for the IS in general. However slightly higher RSDs could be noted for Degeberga compared to the other two locations. The matrix composition at Pynten (Kristianstad) caused a decrease in flow rate at the end of the sample loading procedure when approximately 100 mL of samples were left to process. Raising the pressure to the maximum of 1.5 bar could not compensate for the increased back pressure in the SPE column, and the sample finished with a flow rate of 27 mL/min instead of the starting value set at 40 mL/min.

A total of 11 out of 26 investigated compounds were identified in Degeberga and St Olof, while 15 compounds were found in Pynten (Kristianstad), out of which five compounds displayed concentrations above 100 ng/L—sulfamethoxazole, carbamazepine, diclofenac, metoprolol and oxazepam (marked in grey). RSD values were very low for these real-world recipient samples at all sampling sites. With very few exceptions, RSDs never exceeded 15%. It could be noted that at Degeberga the RSDs deviated a bit more as compared to the results at the other two locations. The two antibiotics, sulfamethoxazole and trimethoprim, were present at all three locations, as were citalopram, erythromycin, estrone, and fluconazole. 

## 3. Discussion

As mentioned in the introduction, commercial literature speaks of recommended and applied flow rates in the development of SPE methods intended for polar and semi-polar organic trace analysis [2,3,4]. This is also true for scientific publications [12]. These flow rate figures are also excluded in the scientific literature [13,14], hence they are not to be considered a crucial factor when SPE methodology is under targeted evaluation [15]. The recommendation is generally not to exceed 10 mL/min, and sometimes recommendations are even less [3]. Only in rare occasions has the flow rate exceeded 10 mL/min as exemplified by Öllers and co-workers, who set the flow rate to 15 mL/min using Waters Oasis HLB SPE cartridges (60 mg, 3 mL) for quantification of neutral and acidic pharmaceuticals and pesticides [16]. 

We were surprised by the results in the recovery experiments of spiked pure water samples as presented in Table 1. Severe breakthroughs were expected at the higher flow rates since the recommended flow rates by several manufacturers exceeded four times at the highest flow rate of 40 mL/min [2,3,4]. On the contrary, the ANOVA analysis showed very clearly that there were no significant differences in absolute recoveries between the investigated flow rates for a vast majority of the compounds. The most likely explanation at this point is probably the fact that flow rates previously have been considered a factor not to impinge upon.

The significantly higher absolute recovery at 40 mL/min for six of the compounds may be interpreted as a result of decreased compound adsorption to the inner wall of the glass bottle as the contact time decreased at the higher flow rate. It is known that untreated glassware contains silicate and silanol groups that can act as ion-exchange and nucleophilic centers [10]. The low recoveries of ciprofloxacin, both as a mother compound and as IS, may be explained by the fact that ciprofloxacin can bind to exchangeable cations (which are bound to negatively charged surfaces) via the β-keto acid structure in the molecule [17].

The trend of generally higher calculated relative recoveries for the lowest flow rate compared with the higher flow rates can be explained by the difference in absolute response expressed by the labeled compounds (Table 1). One could also consider shifting the matchmaking of compound-IS pairs, already at this early point in method development, based on the relative recoveries. For example, citalopram could be better off with an IS that shows less absolute recovery, instead of using carbamazepine_D10 as an IS. 

The noticed general drop in absolute recovery for the spiking experiment in pond water was as expected, due to matrix effects [11]. The low mean RSD of 3.6% at the high-level concentration, must therefore be considered satisfying given the complexity of both matrix and analysis method (Table 2). Doxycycline was an exception, with an RSD of 36.7%. Azithromycin and doxycycline, originally linked to the low recovery of ciprofloxacin, behaved differently in presence of the matrix. Turning to the relative recoveries at both low and high concentration levels (Table 2, low, column 6 and high, column 7), it is clear that for a majority of the compounds these are very good, meaning that in most cases the choice of IS works well (Table 1, IS standard, column 15) in the presence of matrix. For a few compounds, the relative recoveries are somewhat too high or low though. Some of the more striking examples of high relative recoveries at the high concentration level are azithromycin (382.4%) and doxycycline (436.7%), both originally linked to the low recovery of the IS ciprofloxacin. Two other examples of high relative recoveries at the high concentration level are triallate (182.1%) and 2-ethylhexyl 4 methoxycinnamate (224.5%), both combined with carbamazepine_D8, for which recombination of IS could be enough. The large relative recoveries of doxycycline and azithromycin would most likely be avoided by allowing them to have their own labeled IS. At the other end of the scale, there is also amoxicillin, which has a relatively low recovery of 44.6%, that possibly could gain from its own labeled IS.

There are many scientific publications dealing with analyzing these types of waters, so the individual compound results in absolute figures will not be discussed in detail. We can conclude, though, that the produced concentration levels (Table 3) and findings are in line with what other researchers have found in recipients affected by STP effluents [18]. Petrovic argued that for a multimethod, recovery outside of the required range of 70%–130% is acceptable if the precision is good and RSD is 20% [8]. All three investigated waters procedures had RSDs < 20%, regardless of the concentration of the reported compound. In Pynten (Kristianstad), where the concentrations were relatively high, RSDs were very low, and in parity with those observed for spiked matrix-rich pond water. This is expected since there is always an error margin in the analysis, which will have a greater impact on a low concentration number. The concentration results from Degeberga showed somewhat higher RSDs than St Olof, despite the concentrations being in the same order of magnitude. When examining the individual samples from Degeberga, it became clear that sample 1 had a somewhat deviating internal standard response (data not shown). This could probably be caused by a somewhat increased amount of labeled standard added to this sample, and as a gentle reminder of the importance of humbleness towards organic trace analysis.

## 4. Materials and Methods

**Figure 3:**
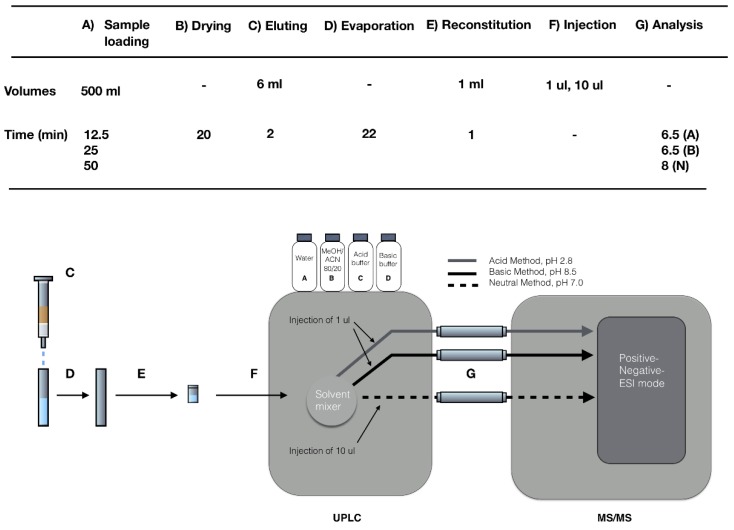
Schematic layout of the entire analytical procedure to put the sample preparation steps of the analytical chain in context, and to give a brief summary of the UPLC MS/MS system setup.

As this work’s primary focus was to test the hypothesis that flow rate can be increased without compromising analytical recovery in environmental analysis, detailed method descriptions will only be given for the SPE loading procedure. In Figure 3, a schematic layout is presented to put the sample loading procedure in the analytical chain in context, and to give a brief summary of the UPLC MS/MS system set up. As time and volume are key parameters in environmental chemistry analysis, these variables are listed for the individual analytical steps. The analytical chain is relatively straight forward between elution (C) and injection (F). The required part of the final analysis is comprised of a unique UPLC MS/MS setup. In short, the UPLC MS/MS (G) analysis was built up around three individual chromatographic methods; acid (A), basic (B), and neutral (N). During method development, each compound was evaluated and optimized without prejudices regarding historical residence in terms of chromatographic conditions and ESI (Electrospray ionization) mode. This unique strategy embraces the full potential of a multi-component UPLC-ESI-MS/MS method adapted to cover emerging contaminants of many different polarities, minimizing the elements of compromise in the performance of the final analytical separation and detection. Both sample preparation and final analysis is described in detail (chemicals, preparations, instrumentation, and quantification) in [19,20,21]. 

In Table 1 and column 16, each compound’s original method belonging is noted as acid, basic or neutral. A fraction (1 µL or 10 µL) of each prepared sample vial volume (F) was injected once in each of the three methods. First, the basic method was applied to all samples, then the acid method, and finally the neutral method. One sample faced a total analysis time of 6.5 + 6.5 + 8 min, which included washing and equilibration between individual injections. Every method has its own dedicated UPLC column, as shown in Figure 3.

### 4.1. Chemicals

We used fine ground sand, extra pure, low iron, 40­x100 mesh, from Fischer Scientific (Gothenburg, Sweden). Methanol HPLC-grade was from Sigma–Aldrich (Steinheim, Germany). Ultra-pure water (18.2 MOhm) was obtained from an OPTIMA water purification system (Elga Ltd., High Wycombe, Buckinghamshire, UK). SPE cartridges HLB Oasis (200 mg, 6 mL) were purchased from Waters (Gothenburg, Sweden). 

### 4.2. Instrumentation

#### 4.2.1. Sample Loader

Clean pressurized air was produced by an Atlas Copco SF2 Oil-free air system (Atlas Copco Airpower n.v. B, Wilrijk, Belgium). A flow meter measured the flow rate (Global Quality Analytical Resources, Scantec, Sweden). Two pressure regulators were installed and set to operate at a maximum 1.5 bar. VICI-Cap GL45, equipped with two ports, were used as sample bottle lids (VICI Jour, Schenkon, Switzerland). A Micro Metering Valve Assy 1/8 (IDEX Health & Scientific, Oak Harbor, WA, USA) controlled the flow rate. PTFE tubing (1/8’’, 2.40 mm ID), PTFE Ferrules 1/8’’, Nut, PPS (flangeless) 1/8’’ connector and SPE-adapters were provided by Scantec, Partille, Sweden. DURAN^®^ laboratory bottle, pressure plus+ is recommended (500 mL), Maintz, Germany. A wastewater container and gasket PTFE-tape were purchased at Biltema, Kristianstad, Sweden. SPE-column. 

#### 4.2.2. Sample Loader Construction

The sample loader parts described above were assembled according to the schematics in Figure 1. Two PTFE-tubings of 50 cm each were connected to the two holes in the lid (Figure 1a). One of the tubes is dedicated for air supply and the other tubing will transfer the sample to the SPE-column. The end of this tubing is expanded, hindering it from popping out as pressure is applied, by screwing a screw a couple of turns into the tubing. A total of 2.0 g of finely ground sand was put on top of the SPE column frit. This sand will efficiently hinder larger particles from clogging the SPE inlet frit, enabling efficient whole water analysis. Gasket tape was applied around the SPE- adapter to prevent leakage, and then the adapter, joint with the VICI-lid, and the SPE-column were assembled according to Figure 1a. 

### 4.3. Methods

The three separate experiments (4.3.1, 4.3.2, and 4.3.3) shared the same sample preparation, sample loader construction, sample loader operation, and drying procedure. The samples from these experiments were eluted, reconstituted, and analyzed in the same fashion: 500 g of sample water were weighed in to a 500 mL sample bottle (Figure 1), and 100 µL FoA (10%) and 100 µL EDTA (sat. sol.) was added together with 30 µL of IS mixture. All experiments were carried out in triplicates. 

#### 4.3.1. Recovery from Pure Water at Three Flow Rates 

The investigated flow rates were 10, 20, and 40 mL/min. Samples were prepared according to 4.3. A total of 100 µL of compound mixture was added at high level (Table 2). 

#### 4.3.2. Method Validation. Recovery from Matrix Containing Pond Water at High Flow Rate

The flow rate was set to 40 mL/min. The pond water used was diluted 1:5 in pure water and was estimated to have a humic carbon content of 2.0 mg/L. The carbon content was determined using the UV_254_ method and ERM-CA 100 as reference (carbon content of 20 mg/L).

Samples were prepared according to 4.3. A total of 100 µL of compound mixture was added at low and high levels (Table 2).

#### 4.3.3. Applying the Method to Recipient Samples from Three Different Natural Locations

Samples were collected from three different locations in Scania, Sweden with the following coordinates; St Olof: 55°38’25.3”N 14°8’3.3E, Degeberga: 55°49’47.4”N 14°7’12.0”E, and Kristanstad: 56°0’50.3”N 14°11’9.8”E. Coordinates are given in the format of the World Geodetic System 1984 (WGS 84). Each sample set triplicate was prepared according to 4.3. An overview of the sampling locations is seen in Figure S1.

#### 4.3.4. Sample Loader Operation

IMPORTANT: Always use two pressure regulators to ensure a safe operation, keeping pressure within the safety margins as described by the glass manufacturer [22]. The bottle used must always: (1) be visually inspected to check that it is in good condition, and (2) as a safety measure placed in a shatterproof container. 

Pressure was set to <0.5 bar. Each of the three trials were executed in the exact same way: flow was measured and set to the desired rate by connecting the flow meter to each SPE outlet, and pressure was adjusted by gently increasing the pressure and fine-tuning by adjusting the Micro Metering Valve (Figure 1a). The flow was monitored at regular intervals during the sample loading procedure, and pressure was adjusted if needed. 

#### 4.3.5. Drying Procedure, Eluting, Reconstituting, and Final Analysis

After each round, pressure was relieved and the SPE-columns brought to drying, according to Figure 1b. A flow of pure air at 2 bar for 30 min dried the columns. The columns were eluted into glass tubes with 6 mL MeOH, and then evaporated at 40 °C for 22 min in a TurboVap, Biotage. The samples were reconstituted in 100 µL MeO, 5 µL EDTA, and 895 µL H_2_O, then analyzed according to the UPLC MS/MS methods described in [19,20,21].

### 4.4. Calculations

#### 4.4.1. Absolute Recovery

Absolute recovery results were calculated as:

Absolute recovery (%) = 100·(a/b)(1)

where a is the MS/MS response of a given compound and b is the MS/MS mean response, generated from four injections from two individual, high-level standards. 

#### 4.4.2. Relative Recovery

The relative recovery results were calculated as described in the comprehensive analytical methodology by the United States Environmental Protection Agency; EPA Method1694 (U.S. Environmental Protection Agency 2007) [20]. The relative response (RR) (labeled to native) vs. concentration in the calibration solutions was computed over the calibration range according to (2):

RR = (A_n_C_l_)/(A_l_C_n_) (2)

A_n_ = the area of the daughter m/z for the native compound
A_l_ = the area of the daughter m/z for the labeled compound
C_l_ = the amount of the labeled compound in the calibration standard (pg)
C_n_ = the amount of the native compound in the calibration standard (pg)

The concentration of a native compound was calculated according to (3):

C_n_ = (A_n_C_l_)/(A_l_RR) (3)

Each pair of compound-IS are listed in Table 1. In experiment 4.3.1 and 4.3.2, relative recoveries are presented as percentage figures of the determined amounts of the added compounds (Table 1 and Table 2). In experiment 4.3.3, the relative recoveries are presented as the determined concentration in ng/L for the natural location samples using IS-method (Table 3). 

#### 4.4.3. ANOVA 

One-way ANOVA were calculated for the means of the absolute recovery in experiment 4.3.1 (Table 1, column 7). F-crit 5.14, at *p* = 0.05. Calculations were made in Excel version 16.21.1. 

## 5. Conclusions

The use of an innovative positive pressure sample loading technique with an in-line filter of finely ground sand, enabled large volume sample loading at a high flow rate with good repeatability. All three flow rates produced highly repeatable results, and this allowed flow rate to increase up to 40 mL/min for a 200 mg, 6 mL, SPE cartridge of reversed phase type without compromising the recoveries. There is, at least as far as for the investigated compounds, a generous flow rate window from 10 to 40 mL/min where sample loading can be conducted. The high flow rate at 40 mL/min is more than four times those previously used, and could mean a substantial contribution for reducing the sample preparation bottleneck. The use of this technology together with the finding of a generous flow rate window allows the sample to (1) be transferred to the SPE-column at the sample location and (2) by the person taking the sample. This further means that only the sample cartridge would be needed to be sent to the laboratory, instead of the whole water sample, as of today’s technique. This approach avoids the degradation that can occur in the transported frozen samples, as sample that is transferred to the sample cartridge as early as possible is much more stable. It also saves the cumbersome handling of the frozen samples. Altogether, these improved sample handling steps may also contribute to a greener analytical protocol by a much more simplified overall procedure [23]. It should also be noted that the novel technology can be appropriate for detection techniques other than MS/MS.

## Figures and Tables

**Table 1:** Absolute and relative recoveries of 26 compounds and 9 isotope labeled standards (IS) spiked in 0.5 liter pure water at a high concentration level applying three different flow rates; A = 10 mL/min, B = 20 mL/min, and C = 40 mL/min. Each experiment (A, B, and C) ran as triplicate. One-way ANOVA was performed on the absolute recovery results at *p* = 0.05, F_crit_ = 5.1. Absolute recovery for 6 compounds (grey marked) exceeded F_crit_ = 5.1. Compounds showing relative recovery lower than 50% are marked in light grey. The absolute amounts added ranged from 10–1000 ng depending on the compound (see Table 2, high, column 10). *Compound is run in both acid and basic UPLC (Ultra-Performance Liquid Chromatography) method. The 9 IS are indicated in italic.

Compound	Absolute Recovery							Relative Recovery							
	A (%)	RSD (%)	B (%)	RSD (%)	C (%)	RSD (%)	ANOVA, F	A (%)	RSD (%)	B (%)	RSD (%)	C (%)	RSD (%)	IS standard	Method
Acetamiprid	97.4	2.4	100.1	1.3	98.4	0.8	1.9	107.9	9.4	101.2	2.5	94.6	3.3	Carbamazepine_D10	Basic
Amoxicillin	3.3	0.3	3.2	0.1	3.1	0.4	0.4	15.7	2.7	10.7	0.7	8.3	1.8	Ciprofloxacin_D8	Acid
Azithromycin	3.5	1.7	4.0	0.4	9.6	0.8	28.1	19.9	11.8	15.0	2.4	27.8	3.5	Ciprofloxacin_D8	Acid
Carbamazepine	93.2	3.6	93.4	2.5	92.0	0.7	0.3	103.0	5.5	94.5	4.7	88.5	1.8	Carbamazepine_D10	Basic
*Carbamazepine_D10*	*90.7*	*8.1*	*98.9*	*3.5*	*104.0*	*2.8*	*4.7*								Basic/Acid*
Ciprofloxacin	22.7	4.1	26.9	3.3	32.2	6.8	2.7	105.2	8.0	89.9	2.7	84.1	11.6	Ciprofloxacin_D8	Acid
*Ciprofloxacin_D8*	*21.4*	*2.2*	*29.8*	*3.2*	*37.9*	*3.1*	*24.6*								*Acid*
Citalopram	53.5	3.8	57.2	6.6	55.4	5.4	0.3	51.9	1.4	51.5	6.1	48.2	5.9	Carbamazepine_D10	Acid
Clarithromycin	55.5	4.3	61.1	1.7	63.2	5.2	3.0	101.4	8.0	94.0	3.3	89.1	2.6	Clarithromycin_D3	Basic
*Clarithromycin_D3*	*54.7*	*0.7*	*65.0*	*2.9*	*70.9*	*4.8*	*19.2*								*Basic*
Clothianidin	101.9	1.6	101.5	4.1	102.3	1.8	0.1	99.1	6.9	91.4	5.5	89.0	2.3	Carbamazepine_D10	Acid
Diclofenac	80.8	2.9	84.7	7.1	85.9	5.9	0.7	104.0	6.1	97.2	2.4	91.1	2.5	Diclofenac_C6	Basic/Acid*
*Diclofenac_C6*	*77.9*	*6.9*	*87.1*	*5.3*	*94.4*	*8.3*	*4.3*								*Basic*
Doxycycline	77.4	1.8	83.6	3.6	76.6	9.2	1.3	75.3	4.2	75.3	2.0	66.8	9.7	Ciprofloxacin_D8	Acid
Erythromycin	21.2	0.1	27.8	1.4	37.7	3.4	45.5	38.7	0.5	42.7	0.7	53.2	4.0	Clarithromycin_D10	Basic
17-Beta-estradiol (E2)	57.6	2.5	62.6	7.2	63.1	3.4	1.2	101.5	6.7	95.7	4.9	90.1	1.7	17-Beta-estradiol (E2)_D5	Neutral
*17-Beta-estradiol (E2)_D5*	*56.9*	*6.0*	*65.3*	*5.2*	*70.0*	*5.0*	*4.5*								*Neutral*
Estrone (E1)	57.0	2.9	62.3	8.0	61.6	4.3	0.8	102.3	7.1	96.1	3.6	89.3	1.7	Estrone (E1)_D4	Neutral
*Estrone (E1)_D4*	*56.0*	*6.4*	*64.8*	*6.8*	*69.0*	*6.0*	*3.2*								*Neutral*
17-Alpha-ethinylestradiol (EE2)	52.2	2.9	59.1	8.7	57.5	5.4	1.1	93.6	6.0	91.1	5.2	83.5	3.1	Estrone (E1)_D4	Neutral
Fluconazole	96.4	0.7	96.3	1.1	97.7	0.8	2.2	106.9	9.3	97.4	3.4	93.9	2.6	Carbamazepine_D10	Basic
Imidacloprid	97.3	0.2	98.0	1.2	97.9	1.7	0.3	107.8	10.4	99.2	2.8	94.2	2.0	Carbamazepine_D10	Basic
Metaflumizone	64.1	12.3	69.9	5.2	89.2	16.5	3.5	84.1	9.3	80.0	2.1	98.0	13.8	Diclofenac_C6	Acid
Methiocarb	68.2	4.0	69.8	5.6	69.4	3.1	0.1	102.0	6.0	94.0	5.5	87.4	3.6	Methiocarb_D3	Basic
*Methiocarb_D3*	*67.1*	*6.5*	*74.3*	*4.2*	*79.5*	*5.3*	*3.9*								*Basic*
Metoprolol	88.8	2.3	90.9	1.5	89.6	3.4	0.5	98.8	4.0	93.4	4.2	89.8	6.0	Metoprolol_D7	Basic
*Metoprolol_D7*	*90.0*	*3.5*	*97.5*	*4.8*	*99.9*	*6.1*	*3.3*								*Basic*
2-Ethylhexyl 4-methoxycinnamate	16.5	4.9	16.2	0.6	29.0	7.5	6.0	15.9	4.1	14.6	0.4	25.3	7.1	Carbamazepine_D10	Acid
Oxadiazone	48.8	3.9	53.3	4.1	52.9	8.8	0.5	47.3	1.3	48.0	3.9	46.1	8.7	Carbamazepine_D10	Acid
Oxazepame	85.7	2.5	87.0	4.1	86.7	3.4	0.1	110.4	7.0	100.0	3.1	92.1	4.3	Carbamazepine_D10	Basic
Sulfamethoxazole	96.2	1.8	95.5	0.4	93.5	3.2	1.2	102.1	6.8	94.0	3.4	88.0	3.3	Sulfamethoxazole_C6	Acid
*Sulfamethoxazole_C6*	*94.4*	*4.8*	*101.6*	*3.8*	*106.3*	*0.8*	*8.5*								*Acid*
Thiamethoxam	100.2	1.3	101.4	0.7	100.8	1.1	1.0	111.0	9.2	102.6	3.1	97.0	3.6	Carbamazepine_D10	Basic
Triallate	9.9	0.7	9.8	0.9	10.4	2.4	0.1	55.7	11.4	36.8	4.9	29.5	2.4	Carbamazepine_D10	Acid
Trimethoprim	88.3	0.9	87.2	0.2	86.1	1.4	3.9	97.9	9.6	88.2	3.0	82.9	3.5	Carbamazepine_D10	Basic
**Mean:**	64.2	3.3	68.2	3.5	70.7	4.3	F-crit 5.14	83.1	6.6	76.7	3.3	74.1	4.5		

**Table 2:** Absolute and relative recoveries of 26 compounds spiked in 0.5 liter diluted matrix-rich pond water (1:5) at high and low concentration levels applying a flow rate of 40 mL/min. The experiment ran as a triplicate. The absolute amount (ng) of compounds spiked to the sample at low and high levels are shown in column 9 and 10. The absolute recovery is calculated for the high-level values as in the previous experiment (Table 1). Compounds are ranked based on absolute recovery, highest to lowest. The 9 IS are indicated in italic. Six compounds showed higher absolute recovery in the presence of matrix and two of them exceeded 100 %, grey marked. Four compounds showed excessive relative recoveries, light grey.

	Absolute Recovery		Aq-Matrix	Relative Recovery					
Compound	High (%)	RSD (%)	Difference	Low (%)	RSD (%)	High (%)	RSD (%)	Low (ng)	High (ng)
Doxycycline	198.3	36.7	−121.7	30.4	2.3	436.7	97.8	5.0	50.0
Azithromycin	107.0	2.7	−97.4	87.1	0.7	382.4	16.6	5.0	50.0
Clothianidin	86.7	2.8	2.3	98.9	2.2	125.8	3.7	1.0	10.0
Sulfamethoxazole	82.3	2.4	11.2	116.7	0.4	119.3	4.0	1.0	10.0
Clarithromycin	65.9	3.6	−2.7	78.3	0.5	105.8	3.8	1.0	10.0
Carbamazepine	64.5	0.7	27.5	122.4	0.4	127.9	6.2	1.0	10.0
Metoprolol	63.4	1.6	26.2	94.4	0.5	100.7	2.9	1.0	10.0
Erythromycin	62.6	3.6	−24.9	112.7	0.2	100.6	4.9	1.0	10.0
Trimethoprim	60.8	1.4	25.4	125.9	0.0	120.4	5.2	1.0	10.0
Acetamiprid	60.7	1.0	37.6	119.8	0.3	120.4	4.9	1.0	10.0
Imidacloprid	60.0	2.5	38.0	116.1	0.7	118.9	1.8	1.0	10.0
Thiamethoxam	58.0	1.3	42.8	105.4	0.2	115.0	3.9	1.0	10.0
Oxazepame	56.9	1.6	29.8	132.8	0.2	112.8	3.8	1.0	10.0
Triallate	50.9	3.9	−40.5	92.5	0.6	182.1	17.2	1.0	10.0
Methiocarb	50.5	2.5	18.9	91.0	0.2	102.0	6.6	1.0	10.0
Oxadiazone	50.3	2.4	2.6	91.9	0.6	110.3	10.5	2.0	20.0
Diclofenac	42.3	3.0	43.6	115.4	1.3	105.1	2.3	1.0	10.0
2-Ethylhexyl 4-methoxycinnamate	38.9	9.5	−9.9	119.3	1.7	224.5	59.3	5.0	50.0
Metaflumizone	37.8	4.4	51.4	66.2	0.2	61.9	8.2	5.0	50.0
Citalopram	36.7	1.8	18.6	68.3	0.1	80.7	8.0	1.0	10.0
17-Beta-estradiol (E2)	34.8	1.1	28.3	102.3	0.8	105.6	7.1	50.0	500.0
Fluconazole	34.3	1.2	63.3	65.0	0.1	68.0	1.6	1.0	10.0
17-Alpha-ethinylestradiol (EE2)	31.6	1.0	25.9	91.9	0.9	100.4	5.5	100.0	1000.0
Ciprofloxacin	30.1	3.3	0.12	26.6	0.6	108.4	13.5	5.0	50.0
Estrone (E1)	28.8	1.2	32.8	88.8	0.3	91.7	2.7	10.0	100.0
Amoxicillin	1.3	0.3	1.9	137.6	3.1	44.6	9.8	10.0	100.0
*Sulfamethoxazole_C6*	*68.5*	*4.0*	*37.8*						
*Metoprolol_D7*	*62.8*	*2.9*	*37.1*						
*Clarithromycin_D3*	*62.1*	*4.7*	*8.8*						
*Carbamazepine_D10*	*50.4*	*2.8*	*53.6*						
*Methiocarb_D3*	*49.5*	*3.2*	*29.9*						
*Diclofenac_C6*	*40.2*	*2.1*	*54.3*						
*17-Beta-estradiol (E2)_D5*	*33.0*	*2.8*	*37.0*						
*Estrone (E1)_D4*	*31.4*	*2.2*	*37.6*						
*Ciprofloxacin_D8*	*27.4*	*0.5*	*0.01*						
**Mean:**	54.7	3.6							

**Table 3:** Results for the 26 investigated compounds in recipient samples at three different natural locations: Degeberga, St Olof and Pynten. Each recipient water sample was previously processed in triplicates applying a flow rate of 40 mL/min. The results are presented based on findings in Pynten (close to the city of Kristianstad), ranging from the highest to lowest concentration. Five compounds displayed concentrations above 100 ng/L, light grey. The sampling locations can be seen in Figure S1.

	Absolute Recovery						Relative Recovery					
Compound	Degeberga (%)	RSD (%)	St Olof (%)	RSD (%)	Pynten (%)	RSD (%)	Degeberga (%)	RSD (%)	St Olof (%)	RSD (%)	Pynten (%)	RSD (%)
Acetamiprid							nd	-	nd	-	0.2	13.9
Thiamethoxam							nd	-	nd	-	0.9	23.9
Estrone (E1)							0.1	3.6	0.7	2.2	2.2	5.5
Imidacloprid							0.2	12.8	nd	-	3.0	2.3
Clarithromycin							nd	-	0.04	2.9	12.6	4.9
Azithromycin							nd	-	nd	-	15.8	1.4
Fluconazole							1.1	13.0	0.5	9.5	29.7	3.8
Citalopram							2.7	17.0	3.4	3.4	58.0	7.5
Erythromycin							1.2	11.9	0.7	9.7	62.7	2.5
Trimethoprim							0.2	3.6	3.6	1.7	75.2	4.9
Sulfamethoxazole							2.5	12.3	4.8	3.5	124.0	4.4
Carbamazepine							79.1	12.7	0.6	10.3	224.3	3.2
Diclofenac							30.3	13.1	20.7	1.8	374.5	3.5
Metoprolol							2.5	10.7	33.6	3.0	422.5	5.1
Oxazepame							20.8	14.8	14.1	6.8	473.0	4.1
Amoxicillin							nd	-	nd	-	nd	-
Ciprofloxacin							nd	-	nd	-	nd	-
Clothianidin							nd	-	nd	-	nd	-
Doxycycline							nd	-	nd	-	nd	-
17-Beta-estradiol (E2)							nd	-	nd	-	nd	-
17-Alpha-ethinylestradiol (EE2)							nd	-	nd	-	nd	-
Metaflumizone							nd	-	nd	-	nd	-
Methiocarb							nd	-	nd	-	nd	-
2-Ethylhexyl 4-methoxycinnamate							nd	-	nd	-	nd	-
Oxadiazone							nd	-	nd	-	nd	-
Triallate							nd	-	nd	-	nd	-
Carbamazepine-D10	63.6	8.1	51.9	0.8	33.8	1.1						
Ciprofloxacin_D8	7.8	0.6	4.3	2.1	3.1	1.1						
Clarithromycin_D3	51.5	5.6	47.8	1.8	60.7	2.3						
Diclofenac_C6	51.3	8.0	40.2	3.3	30.1	1.1						
17-Beta-estradiol (E2)_D5	42.2	4.6	39.9	2.2	38.6	2.0						
Estrone (E1)_D4	40.5	4.7	39.3	3.1	32.1	1.1						
Methiocarb_D3	62.5	8.0	57.2	4.8	48.0	1.1						
Metoprolol_D7	64.9	7.2	55.7	0.8	41.5	1.8						
Sulfamethoxazole_C6	99.7	12.8	70.9	3.6	45.7	1.8						

## Supplementary Materials

Figure S1: Overview of the three different natural sampling locations at Pynten (Kristianstad), Degeberga, and St Olof.

## References

[1] Petrovic M., Farré M., de Alda M.L., Pérez S., Postigo C., Köck M., Radjenovic J., Gros M., Barcelo D. (2010). Recent trends in the liquid chromatography-mass spectrometry analysis of organic contaminants in environmental samples. J. Chromatogr. A.

[2] (2013). QuickStart Guide to SPE.

[3] (1998). Guide to Solid Phase Extraction. Bulletin 910.

[4] (2015). Beginners Guide to SPE.

[5] Lopez-Serna R.L., Petrovic M., Barcelo D. (2011). Development of a fast instrumental method for the analysis of pharmaceuticals in environmental and wastewaters based on ultra high performance liquid chromatography (UHPLC)-tandem mass spectrometry (MS/MS). Chemosphere.

[6] (2015). COMMISSION IMPLEMENTING DECISION (EU) 2015/495 of 20 March. Establishing a Watch List of Substances for Union-Wide Monitoring in the Field of Water Policy Pursuant to Directive 2008/105/EC of the European Parliament and of the Council. L 78/40.

[7] (2018). COMMISSION IMPLEMENTING DECISION (EU) 2018/840 of 5 June 2018. Establishing a Watch List of Substances for Union-Wide Monitoring in the Field of Water Policy Pursuant to DIRECTIVE 2008/105/EC of the European Parliament and of the Council. L 141/61.

[8] Petrovic M. (2014). Methodological challenges of multi-residue analysis of pharmaceuticals in environmental samples. Trends Environ. Anal. Chem..

[9] Svahn O., Björklund E. (2019). Simple, fast and inexpensive large “whole water” volume sample SPE-loading using compressed air and finely ground sand. Anal. Methods VL IS.

[10] Suri R.P.S., Singh T.S., Chimchirian R.F. (2011). Effect of process conditions on the analysis of free and conjugated estrogen hormones by solid-phase extraction–gas chromatography/mass spectrometry (SPE–GC/MS). Environ. Monit. Assess..

[11] Wickramasekara S., Hernández-Ruiz S., Abrell L., Arnold R., Chorover J. (2012). Natural dissolved organic matter affects electrospray ionization during analysis of emerging contaminants by mass spectrometry. Anal. Chim. Acta.

[12] Petrie B., Youdan J., Barden R., Kasprzyk-Hordern B. (2016). Multi-residue analysis of 90 emerging contaminants in liquid and solid environmental matrices by ultra-high-performance liquid chromatography tandem mass spectrometry. J. Chromatogr. A.

[13] Gracia-Lor E., Martínez M., Sancho J.V., Peñuela G., Hernández F. (2012). Multi-class determination of personal care products and pharmaceuticals in environmental and wastewater samples by ultra-high performance liquid-chromatography-tandem mass spectrometry. Talanta.

[14] Hladik M.L., Kolpin D.W., Kuivila K.M. (2014). Widespread occurrence of neonicotinoid insecticides in streams in a high corn and soybean producing region. USA. Environ. Pollut..

[15] Baker D.R., Kasprzyk-Hordern B. (2011). Critical evaluation of methodology commonly used in sample collection. storage and preparation for the analysis of pharmaceuticals and illicit drugs in surface water and wastewater by solid phase extraction and liquid chromatography-mass spectrometry. J. Chromatogr. A.

[16] Öller SSinger H.P., Fässler P., Müller S.R. (2001). Simultaneous quantification of neutral and acidic pharmaceuticals and pesticides at the low-ng/l level in surface and waste water. J. Chromatogr. A.

[17] Nowara A., Burhenne J., Spiteller M. (1997). Binding of Fluoroquinolone Carboxylic Acid Derivatives to ClayMinerals. J. Agric. Food Chem..

[18] Pérez-Fernández V., Rocca L.M., Tomai P., Fanali S., Gentili A. (2017). Recent advancements and future trends in environmental analysis: Sample preparation, liquid chromatography and mass spectrometry. Anal. Chim. Acta.

[19] Svahn O., Björklund E. (2016). Increased electrospray ionization intensities and expanded chromatographic possibilities for emerging contaminants using mobile phases of different pH. J. Chromatogr. B.

[20] (2007). Method 1694: Pharmaceuticals and Personal Care Products in Water, Soil, Sediment, and Biosolids by HPLC/MS/MS.

[21] Svahn O. (2016). Tillämpad Miljöanalytisk Kemi för Monitorering Och Åtgärder av Antibiotika- Och Läkemedelsrester i Vattenriket.

[22] DURAN, Group. http://www.duran-group.com/en/products-solutions/laboratory-glassware/products/laboratory-glass-bottles/.

[23] Płotka-Wasylka J. (2018). A new tool for the evaluation of the analytical procedure: Green Analytical Procedure Index. Talanta.

