# Supplementary figures and images for: High Flow-Rate Sample Loading in Large Volume Whole Water Organic Trace Analysis Using Positive Pressure and Finely Ground Sand as a SPE-Column In-Line Filter
Source: Molecules. 2019 Apr 11;24(7):1426. doi: 10.3390/molecules24071426 (PMC6479934; doi:10.3390/molecules24071426)

Figure S1

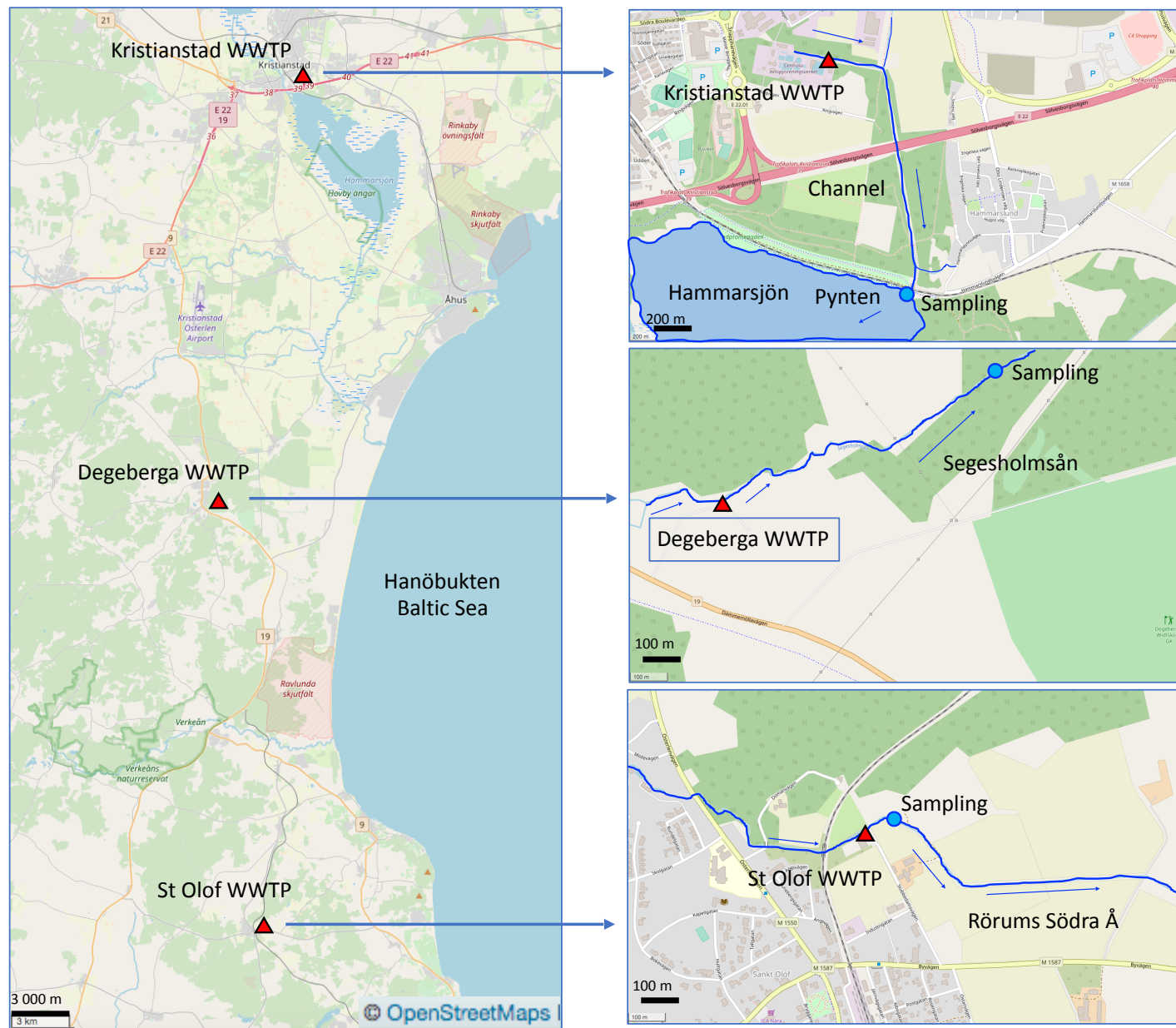

Supplement: Supplementary file 1 [file molecules-24-01426-s001.pdf]
